# Reproductive Immunology and Pregnancy 3.0

**DOI:** 10.3390/ijms242316606

**Published:** 2023-11-22

**Authors:** Dariusz Szukiewicz

**Affiliations:** Department of Biophysics, Physiology & Pathophysiology, Faculty of Health Sciences, Medical University of Warsaw, 02-004 Warsaw, Poland; dariusz.szukiewicz@wum.edu.pl

This Special Issue, the third dedicated to reproductive immunology and pregnancy, is another review of the latest trends in research topics in this field. The dynamic development of immunopathology of the reproductive system creates new opportunities to verify hypotheses regarding the etiopathogenesis of endometriosis and infertility, among other conditions [1].

While estrogens themselves do not cause endometriosis, the disease is classified as estrogen-dependent, in which cells with characteristics very similar to endometrial cells form ectopic foci outside the uterine cavity. Because endometriosis cells may express estrogen receptors (ERα, Erβ, GPER) and progesterone (P4) receptors (PR-A, PR-B), their growth, cyclic proliferation, and breakdown are similar to the processes that occur in the endometrium [2]. This leads to significant complications, mainly due to the chronic inflammatory response. Endometriosis affects 10–15% of women of reproductive age and is associated with chronic pelvic pain, dysmenorrhea, dyspareunia, and infertility [3,4].

The pathogenesis of endometriosis is multifactorial [5,6]. There are several theories, including the widely accepted implantation theory, which assumes the occurrence of retrograde transport of viable endometrial cells with retained abilities for pelvic cavity attachment, proliferation, differentiation, and subsequent invasion into the surrounding tissue [7]. Achievements in the field of immunobiology and embryology have made it possible to supplement implantation theory with knowledge about the significant contribution of stem cells, leading to the development of the stem cell theory of endometriosis [8]. A population of stem cells in the uterus helps the endometrium regenerate after shedding each month during menstruation. These stem cells divide and produce more endometrial tissue to replace what is lost during menstruation. Accordingly, the most abundant cells in the endometrium are endometrial stromal cells (EnSCs) [8,9]. These cells constitute a particular population with clonogenic activity that resembles the properties of mesenchymal stem/stromal cells (MSCs). Thus, a significant role of stem cell-based dysfunction in the formation of initial endometrial lesions is suspected. There is increasing evidence that the role of epigenetic mechanisms and processes in endometriosis has been underestimated [5,10,11,12]. This conclusion is based on the fact that heritable phenotype changes that do not interfere with the DNA sequence are common triggers for hormonal, immunological, and inflammatory disorders, which play a key role in the formation of endometriotic foci [7].

The instability of estrogen/P4 homeostasis, which leads to excessive estrogen exposure and P4 resistance, is strongly reflected in endometriotic tissue as changes in the expression of transcription factors of the estrogen and P4 signaling pathways [9].

It is well known that endometriosis co-occurs in individuals with autoimmune diseases more often than in the general population [13]. This includes individuals with systemic lupus erythematosus (SLE), Hashimoto’s autoimmune thyroiditis, multiple sclerosis (MS), diabetes mellitus type 1, rheumatoid arthritis (RA), Graves’ disease, vitiligo, and celiac disease (CD), among others [13,14,15]. In autoimmune diseases, the immune system mistakenly recognizes its own tissues as immunologically foreign and then induces cellular immunity mechanisms based on T lymphocytes (T cells), macrophages, and natural killer (NK) cells to destroy them. The results of recent studies indicate significant disturbances in the function of these cells in women with endometriosis. For example, an increased number and high activity of regulatory T cells (Tregs) and macrophages are found in the peritoneal fluid of women with endometriosis. In relation to T cells, maintenance of forkhead box P3 (Foxp3) protein—the master regulatory protein involved in Treg-mediated immune system responses—seems crucial to ensure a balanced immune response [16]. Significant differences between autoimmunity and endometriosis in relation to the activity of Tregs and autoreactive effector CD4+ T helper (Th)1 and Th17 subsets are simplified in Figure 1.

Dysfunctional natural killer (NK) cells may also contribute to the impaired recognition of ectopic foci in endometriosis and the lack of effective removal of endometrial cells by the immune system. Reis et al. (2022) [21] reviewed, summarized, and updated the previous literature on NK cells and endometriosis, focusing on the current state of knowledge about the role of NK receptors (NKRs).

The overexpression of NK cell inhibitory receptors (KIRs), such as CD158a+, KIR2DL1, CD94/NKG2A, PD-1, NKB1, and EB6, and inhibitory ligands, namely, PD-L1, HLA-E, HLA-G, and HLA-I, may play an important role in the pathogenesis of endometriosis.

Moreover, the early onset of preeclampsia may also be caused by immune checkpoint disorders, with the presence of a population of NK cells with abnormal KIR expression causing a predisposition to inadequate uterine spiral artery remodeling and shallow trophoblast invasion [22]. It has been shown that immunological interactions in the maternal–fetal system occurring in early pregnancy are not only moderated by T lymphocytes but also by NK cells, which may be because decidual NK cells are the largest population of immune system cells in the uterus during early pregnancy [23]. It has been shown that immunological interactions in the maternal–fetal system occurring in early pregnancy are moderated mainly by NK cells, not T cells, which may be because decidual NK cells are the largest population of immune system cells in the uterus during early pregnancy [23].

Fertility disorders related to abnormal functioning of the immune system, increasing the risk of autoimmune diseases, are a constantly growing and supplemented pool of causes of infertility [24]. Antiphospholipid syndrome (APLS) is characterized by thrombosis and/or recurrent pregnancy loss coexisting with the presence of circulating autoantibodies that are directed against phospholipid-binding proteins (antiphospholipid or aPL antibodies) [25]. Minimal vasculitis combined with complement consumption in patients experiencing infertility may be an underlying mechanism for impaired implantation because aPL antibodies regulate the inflammatory response [26]. Recently, a novel autoantibody against a complex of β2-glycoprotein I and human leukocyte antigen class II molecules (β2-GPI/HLA-DR) has been reported to be an independent autoantibody associated with APLS [27]. In addition, human leukocyte antigen G (HLA-G), expressed on trophoblastic cell surfaces, seems to be one of the main molecules involved in the modulation of both local and systemic maternal immune responses. It was demonstrated that HLA-G 3’UTR polymorphisms and haplotypes may be involved in unexplained recurrent spontaneous abortion (URSA) and may be a predictor of pregnancy outcome [28].

Alpha-enolase (enolase 1, ENO1) is a multifunctional protein that acts as a key glycolytic enzyme in the cytoplasm and a receptor for plasminogen expressed on the cell surface. In euthyroid females with autoimmune thyroiditis, serum levels of autoantibodies against strong epitopes of α-enolase may be treated as good predictive markers for pregnancy loss [29].

Adipokines are cell-signaling molecules (cytokines) produced in adipose tissue and are involved in metabolic, endocrinological, vascular, and immunogenic processes. The obesity pandemic, also occurring among pregnant women, has undoubtedly contributed to the increased interest in the study of adipokines in recent years. Many of these studies aimed to explain the relationship between the concentrations of specific adipokines (e.g., fatty acid binding protein 4, FABP4), especially in pregnant women with weight disorders, and the course of pregnancy and the risk of complications, such as gestational diabetes mellitus (GDM), preeclampsia (PE), fetal growth restriction (FGR), and macrosomia [30,31,32].

The most well-studied gestational trophoblastic disease (GTD) is hydatidiform mole (also known as a molar pregnancy), which is characterized by an overgrown villous trophoblast with cystic “swollen” villi that develop inside the uterus after conception. The peculiarity of this rare disease, which occurs with distinct geographical variations in approximately 1 in 1200 pregnancies, is that the tumor essentially originates from the pregnancy tissue and not from the mother’s tissue [33]. A mole is classified as partial when the cells are triploid and contain two copies of the paternal genome and one copy of the maternal genetic material, and—in the absence of the maternal genome—as complete (contains two sets of the paternal chromosomes) [33]. This feature was used in the differential diagnosis of mole by examining paternally imprinted genes. Paternally imprinted genes are those that are expressed only when inherited from the mother, while the father’s allele is silenced by DNA methylation [34]. Because complete hydatidiform mole lacks a maternal genomic component, there is no expression of paternally imprinted genes in this pathological tissue. Since approximately 40 parentally imprinted genes have been identified in humans, the number of genetic markers in complete hydatidiform mole is constantly increasing. To date, the diagnostic utility has been confirmed with immunohistochemistry of cyclin-dependent kinase inhibitor 1C (p57, encoded by CDKN1C imprinted gene), retinoblastoma transcriptional corepressor 1 (RB1), and pleckstrin homology-like domain family A member 2 (IPL/TSSC3) [35,36,37,38,39].

Lactation in mammals completes the reproductive process, although milk production generally interferes with fertility by inhibiting ovulation. In addition to its reproductive functions, prolactin—the main hormone responsible for breast development during pregnancy and lactation—is also involved in metabolism, osmoregulation, immunomodulation, and behavior. Lactation greatly changes the mother’s metabolism, redistributing the blood supply and increasing the demand for nutrients. The lack of a balanced diet in breastfeeding women carries a high risk of macro- and micronutrient deficiencies and makes it difficult to maintain a healthy body weight due to metabolic disorders. In general, apart from the obvious benefits for the baby, lactation also has a positive effect on the body of the breastfeeding mother. Some cancers, type 2 diabetes, high blood pressure, and nonalcoholic fatty liver disease (NAFLD) are less common among women who breastfeed [40]. However, in a situation unique to humans, i.e., industrial milk production in cows, intense and maximally prolonged lactation has an adverse effect mainly on liver function and the immune system. Cheng et al. (2023) [41] demonstrated that the functionality of circulating leukocytes in dairy cows is suppressed after calving, with negative energy balance as a risk factor. Moreover, the leukocytes of multiparous and primiparous cows responded differently to the diets across age, nutrient supply, and immunity, affecting health and subsequent fertility.

## Figures and Tables

**Figure 1:**
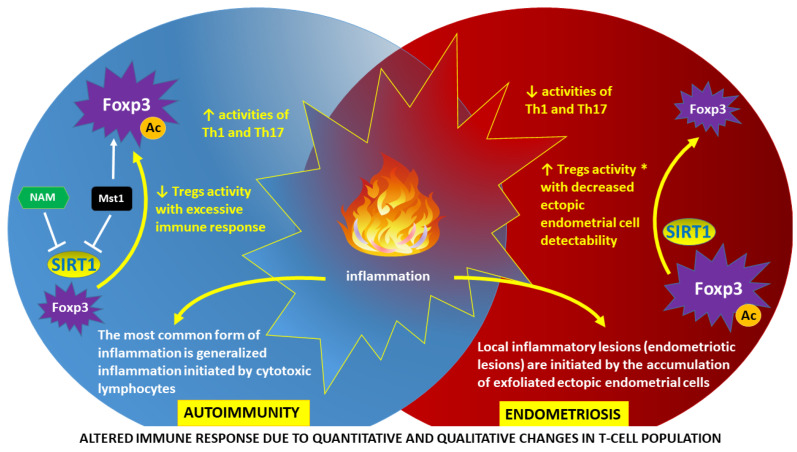
Altered immune response as a result of epigenetically determined dysfunction of regulatory T cells (Tregs): autoimmunity vs. endometriosis. Tregs are essential for maintaining immune homeostasis. Forkhead box P3 (Foxp3) is the master regulatory protein involved in Treg development and function [17]. The activity of Foxp3 is a derivative of the degree of acetylation (Ac), which is directly related to the presence of sirtuin-1 (SIRT1). SIRT1 functions as a nicotinamide adenine dinucleotide (NAD+)-dependent protein deacetylase linked to cellular energy and redox status. Deacetylation of Foxp3 in autoimmunity may be inhibited by nicotinamide (NAM), a byproduct of an SIRT1-catalyzed reaction. Another mechanism of SIRT1 inhibition in autoimmunity involves phosphorylation of SIRT1 by mammalian sterile 20-like kinase 1 (Mst1) protein kinase with subsequent protein p53 acetylation and transactivation, resulting in apoptosis induction and decreased cell proliferation [18]. A high level of Foxp3 acetylation in autoimmunity leads to a decrease in the activity of Tregs and, consequently, to an increase in the activity of autoreactive effector CD4+ T helper (Th)1 and Th17 subsets [19]. Conversely, in endometriosis, a low level of Foxp3 acetylation with uninhibited deacetylating function of SIRT1 leads to an increase in Tregs activity (*) and subsequent reduction in Th1 and Th17 activities [20]. Therefore, the detection of ectopic locations of endometrial cells (endometriotic foci) by the immune system is impaired. An inherent symptom of both autoimmunity and endometriosis is inflammation, although with different pathogenesis and characteristics [16].

## Data Availability

No new data were created or analyzed in this study. Data sharing is not applicable to this article.
